# Interleukin-17 Weakens the NAFLD/NASH Process by Facilitating Intestinal Barrier Restoration Depending on the Gut Microbiota

**DOI:** 10.1128/mbio.03688-21

**Published:** 2022-03-10

**Authors:** Shuying He, Shudan Cui, Wen Song, Yonghong Jiang, Hongsheng Chen, Dongjiang Liao, Xinpeng Lu, Jun Li, Xueqing Chen, Liang Peng

**Affiliations:** a Department of Gastroenterology, First Affiliated Hospital of Guangzhou Medical University, Guangzhou Medical University, Guangzhou, China; b Shenzhen People's Hospital, Shenzhen, China; c Southern Medical University, Guangzhou, China; d State Key Laboratory of Respiratory Disease, National Clinical Research Center for Respiratory Disease, Guangzhou Institute of Respiratory Health, the First Affiliated Hospital of Guangzhou Medical University, Guangzhou Medical University, Guangzhou, China; e Department of Hepatobiliary Surgery, First Affiliated Hospital of Guangzhou Medical University, Guangzhou Medical University, Guangzhou, China; Cornell University

**Keywords:** interleukin-17, microbiota dysbiosis, intestinal barrier, endotoxemia, nonalcoholic steatohepatitis

## Abstract

Interleukin-17 (IL-17) is associated with nonalcoholic fatty liver disease (NAFLD) and gut microbiota, and how IL-17 mediates the NAFLD/nonalcoholic steatohepatitis (NASH) process depending on the gut microbiota is unclear. We found that T helper 17 (T_H_17) cells were decreased in the small intestine in a methionine choline-deficient (MCD) diet-induced NASH model. IL-17-deficient (*Il17^−/−^*) mice showed alterations in intestinal microbiota, including the inhibition of probiotic growth and the overgrowth of certain pathogenic bacteria, and were prone to higher endotoxemia levels and more severe gastrointestinal barrier defects than wild-type (WT) mice. Furthermore, T_H_17 cells were responsible for restoring the intestinal barrier after administration of recombinant IL-17 to *Il17^−/−^* mice or injection of CD4^+^ T cells into a *Rag1^−/−^* mouse model. Additionally, transplantation of the microbiota from WT mice to *Il17^−/−^* mice restored the intestinal barrier. Notably, microbiota-depleted *Il17^−/−^* mice were resistant to MCD diet-induced intestinal barrier impairment. Fecal microbiota transplantation from *Il17^−/−^* mice to microbiota-depleted mice aggravated intestinal barrier impairment and then promoted the development of NASH. Collectively, this study showed that host IL-17 could strengthen intestinal mucosal barrier integrity and reduce dysbiosis-induced intestinal injury and secondary extraintestinal organ injury induced by a special diet.

## INTRODUCTION

Diet-related nonalcoholic fatty liver disease (NAFLD)/nonalcoholic steatohepatitis (NASH) is one of the most common causes of chronic liver disease worldwide (1). Although host genetic factors and dietary composition have been associated with NAFLD, other environmental factors are required to elucidate NAFLD. Increased interest has focused on identifying and understanding the specific role of host genetic factors in shaping gut microbiota and altering the disease process in extraintestinal organs. Interleukin-17A (IL-17A [also named IL-17]), a member of the IL-17 family that has emerged as a crucial proinflammatory cytokine, is typically highly expressed in the liver after high-fat-diet (HFD) feeding and promotes the progression of NASH and liver fibrosis (2–5). Surprisingly, the gut microbiota regulates intestinal T helper 17 (T_H_17) cells, and the IL-17 level was obviously decreased in the small intestine (SI) in a metabolic disease-related NAFLD mouse model after HFD feeding, ultimately contributing to the progression of metabolic disease (4, 6). However, some researchers have reported that IL-17-deficient mice were resistant to diet-induced obesity in a gut microbiota-dependent manner after HFD feeding (7). The inconsistent results indicated that other factors are required to elucidate the interaction between IL-17 and NAFLD.

Further discussion has recently focused on the function of the intestinal barrier, which is related to NAFLD and interacts with luminal contents, including those in the microbiota and microbial products (8, 9). An increase in intestinal permeability allows the translocation of microbiota and microbial products into the portal vein (10, 11), and some studies have found that the infusion of lipopolysaccharide (LPS), a component of Gram-negative bacteria, is associated with an HFD (12). Certain studies have provided evidence that IL-17 maintains barrier integrity and limits excessive permeability through the IL-17 receptor (IL-17R) adaptor protein Act-1 in a murine inflammatory bowel disease (IBD) model (13). These results suggest that IL-17 may exert a protective role against NASH by limiting intestinal permeability, and the gut microbiota may also account for the underlying role of IL-17 in maintaining the intestinal barrier.

To determine whether IL-17 deficit-related gut dysbiosis influences the function of the intestinal barrier and explore the mechanism in the NAFLD/NASH process, we used a methionine choline-deficient (MCD) diet to induce a model of NAFLD/NASH, which results in severe NASH with significant hepatic inflammation and fibrosis. We found that IL-17 deficiency (*Il17^−/−^*) disturbed the intestinal barrier, accompanied by intestinal flora alteration, which enhanced the severity of NAFLD/NASH. 16S rRNA gene amplicon sequencing shows increased pathogenic (*Staphylococcaceae*, *Enterococcaceae*, and *Enterobacteriaceae*) and decreased beneficial microorganisms in the gut microbial community of IL-17-deficient mice after MCD feeding. Fecal microbiota transplantation (FMT) and antibiotic treatment can reconstruct the function of the gut barrier, ultimately alleviating liver injury. We further transferred fecal microbiota samples collected from *Il17^−/−^* mice to microbiota-depleted mice, and intestinal barrier injury and liver lipid accumulation were aggravated. Our results demonstrate that an MCD diet can change the gut microbiota and result in intestinal barrier destruction and NAFLD/NASH development through IL-17. When an MCD diet is consumed, IL-17 seems to play a crucial protective role in the liver gut axis in *Il17^−/−^* mice.

## RESULTS

### T_H_17 cell proportions are altered in the intestine and liver of MCD diet-induced NASH/NAFLD mice.

**FIG 1:**
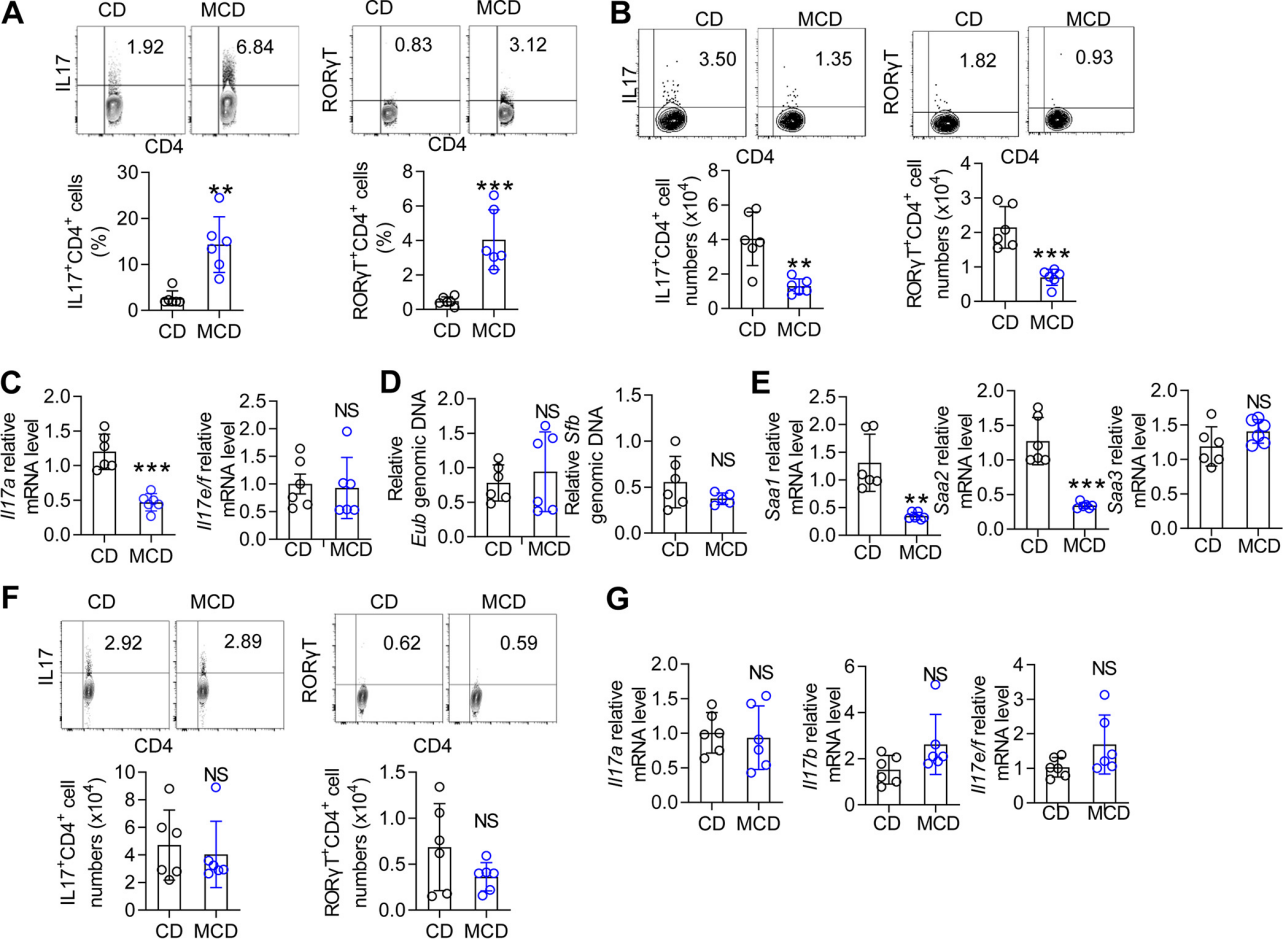
The proportion of T_H_17 cells is changed in MCD diet-fed mice. Mice were fed a CD or an MCD diet for 3 weeks. (A) Flow cytometry analysis of IL-17^+^ CD4^+^ and RORγT^+^ CD4^+^ cells in the liver. (B) Flow cytometry analysis of IL-17^+^ CD4^+^ and RORγT^+^ CD4^+^ cells in the SI. (C) The mRNA levels of *Il17a* and *Il17e*/*f* in the SI were determined by qPCR. (D) SI mucosal DNA was extracted, and qPCR was used to analyze the level of 16S rRNA genes of *Eubacteria*, relative to the level of 18S rRNA genes in mice normalized to biopsy specimen size. The SFB groups were analyzed relative to the level of 16S rRNA. (E) The mRNA levels of *Saa1*, *Saa2*, and *Saa3* in the SI were determined by qPCR. The relative mRNA levels were normalized to that of *Gapdh*. (F) Flow cytometry analysis of IL-17^+^ CD4^+^ and RORγT^+^ CD4^+^ cells in the colon. (G) The *Il17a*, *Il17e*/*f*, and *Il17b* mRNA levels in the colon were determined by qPCR. The relative mRNA levels were normalized to *Gapdh* levels. The data are expressed as means ± standard deviation (SD). Significance versus CD-fed mice: NS, not significant; *, *P* < 0.05; **, *P* < 0.01; ***, *P* < 0.001.

HFD feeding decreases the proportion of T_H_17 cells in the gut and leads to the onset of metabolic disease (4, 6). To confirm whether this phenomenon occurred with other special diets, a NASH model was induced by MCD diet feeding of wild-type (WT) mice. After 3 weeks, the livers of the MCD diet-fed mice exhibited mild steatosis with scattered lobular inflammatory cells (see Fig. S1A in the supplemental material). Next, we analyzed the characteristics of immune cells in the liver (representative fluorescence-activated cell sorter [FACS] gating shown in Fig. S2 in the supplemental material). Compared with chow diet (CD)-fed mice, the MCD diet-fed mice showed an increased proportion of T_H_17 cells (Fig. 1A) and a decreased proportion of FOXP3^+^ regulatory T (Treg) cells in the liver, whereas IFN-γ^+^ T_H_1 cells were not affected by the MCD diet (Fig. S1B and C). We confirmed these results by intracellular staining of the master transcription factor for each T_H_ subset, revealing an increase in RORγT^+^ T_H_17 cells and no change in T-bet^+^ T_H_1 cells (Fig. 1A; Fig. S1B). Similar to previous reports (14), γδ T cells in the liver were also increased by MCD diet feeding, and closer examination of γδ T cell IL-17 production demonstrated a marked increase in the frequency of IL-17^+^ γδ T cells in MCD diet-fed mice (Fig. S1D). The MCD model typically induces liver fibrosis, and CD8 and invariant NKT cells contribute to hepatic fibrosis (15, 16). We characterized the CD8 and invariant NKT cells in the MCD model. Although the CD8 T cells producing IFN-γ were slightly increased, the CD8 T cells and NKT cells remained largely unaltered (Fig. S1E and F).

10.1128/mbio.03688-21.2 FIG S1 Liver immunity is altered by an MCD diet. Mice were fed a CD or an MCD diet for 3 weeks. (A) Representative H&E-stained (upper panel) and Oil Red O-stained (lower panel) liver sections from CD- and MCD diet-fed mice. Scale bar, 50 μm. (B and C) Flow cytometry analysis of IFN-γ^+^ CD4^+^, T-bet^+^ CD4^+^, and FOXP3^+^ CD4^+^ cells in the liver by intracellular staining. (D) Flow cytometry analysis of CD3^+^ γδ TCR^+^ and γδ TCR^+^ IL-17^+^ cells in the liver. (E) Flow cytometry analysis of CD8^+^ CD3^+^ and IFN-γ^+^ CD8^+^ cells in the liver. (F) Flow cytometry analysis of NK1.1^+^ CD3^+^ and IFN-γ^+^ NK1.1^+^ cells in the liver. The data are expressed as means ± SD. Significance versus CD-fed mice: NS, not significant; *, *P* < 0.05; **, *P* < 0.01. Download FIG S1, TIF file, 2.2 MB. Copyright © 2022 He et al. 2022 He et al. https://creativecommons.org/licenses/by/4.0/ This content is distributed under the terms of the Creative Commons Attribution 4.0 International license.

10.1128/mbio.03688-21.3 FIG S2 Representative flow cytometry diagrams of the gating of the various cell populations. (A) Representative flow cytometric analysis of TH cells. (B) Representative flow cytometric analysis of CD8 and NKT cells. (C) Representative flow cytometric analysis of γδ T cells. Download FIG S2, TIF file, 1.2 MB. Copyright © 2022 He et al. 2022 He et al. https://creativecommons.org/licenses/by/4.0/ This content is distributed under the terms of the Creative Commons Attribution 4.0 International license.

Because previous studies suggested that T_H_17 cells usually undergo substantial changes in the SI under HFD feeding conditions (4, 6), we next analyzed the change in the immune cells in the SI. Similar to the previous study findings (4, 6), T_H_17 cells were significantly decreased in lamina propria (LP) lymphocytes of the SI (Fig. 1B), but other T_H_ subsets (Treg and T_H_1 cells) were not affected (see Fig. S3A and B in the supplemental material). IL-17 also originates from γδ T cells or group 3 innate lymphoid cells (ILC3s) in the gut (17). Thus, we also detected ILCs and γδ T cells. The ILC3s in the intraepithelial (IEL) and γδ T cells in the SI LP were not changed by the MCD (Fig. S3C and D). We also analyzed γδ T cells in the IEL zone and found that γδ T cells were increased in MCD diet-fed mice (Fig. S3E). The MCD diet increased IFN-γ^+^ γδ T cells, whereas the IL-17^+^ γδ T cells showed little difference (Fig. S3E). Next, we confirmed that the IL-17A mRNA levels were also decreased in the SI (Fig. 1C). The IL-17 family includes six proteins (IL-17A to IL-17F); we also evaluated the mRNA expression of other IL-17 family members (18). IL-17B mRNA was not detectable, and IL-17E/F was not changed in MCD diet-fed mice compared with that in control mice (Fig. 1C). Segmented filamentous bacteria (SFB) are necessary for robust gut T_H_17 cells (19) and favor T_H_17 cell development because of the release of serum amyloid antigen (SAA) by dendritic cells (DCs) (20). However, the relative abundance of SFB in the SI mucosa of the MCD diet-fed mice was not altered compared with that in CD-fed mice, although the abundance of total bacteria (*Eubacteria*) was unchanged (Fig. 1D). Compared with those in the CD-fed group, the *Saa1* and *Saa2* mRNA levels were also decreased, whereas the *Saa3* levels were not changed (Fig. 1E). These results confirmed that T_H_17 cells were decreased in the SI LP.

10.1128/mbio.03688-21.4 FIG S3 Immunity is altered in the SI of MCD diet-fed mice. Mice were fed a CD or an MCD diet for 3 weeks. (A and B) Flow cytometry analysis of CD4^+^ FOXP3^+^, CD4^+^ IFN-γ^+^, and CD4^+^ T-bet^+^ cells in the SI. (C and D) Flow cytometry analysis of CD3^−^ RORγT^+^ IL-17^+^ (ILC3s) and CD3^+^ γδ TCR^+^ cells in the LP area of the SI. (E) Flow cytometry analysis of CD3^+^ γδ TCR^+^ cells in the IEL area of the SI. The data are expressed as means ± SD. Significance versus CD-fed mice: NS, not significant; *, *P* < 0.05; **, *P* < 0.01. Download FIG S3, TIF file, 1.1 MB. Copyright © 2022 He et al. 2022 He et al. https://creativecommons.org/licenses/by/4.0/ This content is distributed under the terms of the Creative Commons Attribution 4.0 International license.

Finally, we evaluated the immune subgroup in the colon. The T_H_17 cells in the colon LP of the MCD diet-fed mice were not changed compared to those in the colon LP of the control mice (Fig. 1F). Similarly, the *Il17a* mRNA levels were unchanged in MCD diet-fed mice compared with those in control mice (Fig. 1G). Although the *Il17b* and *Il17e*/*f* levels were slightly increased in MCD diet-fed mice, this increase was not statistically significant (Fig. 1G). No difference was found in Treg cells between the MCD diet-fed mice and CD-fed mice, but the T_H_1 cells were significantly increased (see Fig. S4A and B in the supplemental material). γδ T cells were also not affected by the MCD diet in the IEL and LP areas, and ILC3s in the LP of the colon were also not altered (Fig. S4C to E). These data indicated that the intestinal immunity balance was disrupted in the MCD-induced NAFLD/NASH process, and the T_H_17 cell proportion was decreased in the SI but increased in the liver, possibly indicating that T_H_17 cell polarization played an important role in the gut-liver axis in the MCD diet-induced model.

10.1128/mbio.03688-21.5 FIG S4 Immunity is altered in the colons of MCD diet-fed mice. (A and B) Flow cytometry analysis of FOXP3^+^ CD4^+^, IFN-γ^+^ CD4^+^, and T-bet^+^ CD4^+^ cells in the colon. (C and D) Flow cytometry analysis of CD3^−^ RORγT^+^ IL-17^+^ (ILC3s) and CD3^+^ γδ TCR^+^ cells in the LP of the colon. (E) Flow cytometry analysis of CD3^+^ γδ TCR^+^ cells in the IEL of the colon. The data are expressed as means ± SD. Significance versus CD-fed mice: NS, not significant; *, *P* < 0.05; **, *P* < 0.01. Download FIG S4, TIF file, 0.8 MB. Copyright © 2022 He et al. 2022 He et al. https://creativecommons.org/licenses/by/4.0/ This content is distributed under the terms of the Creative Commons Attribution 4.0 International license.

### *Il17* deletion promotes the development of NAFLD/NASH.

**FIG 2:**
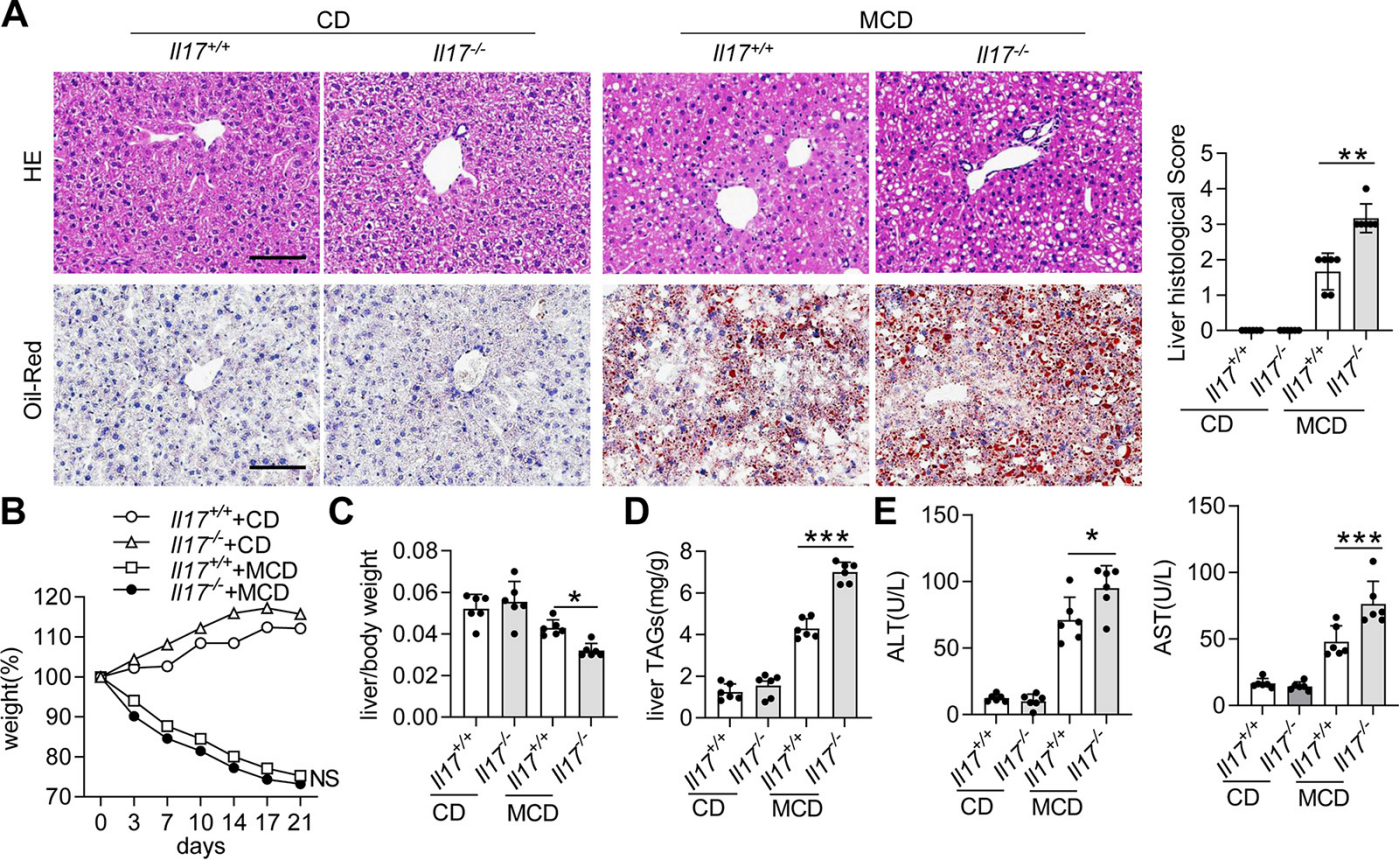
*Il17* deletion aggravates hepatic steatosis. *Il17^+/+^* and *Il17^−/−^* mice were fed a CD or an MCD diet for 3 weeks (*Il17^+/+^*/CD, *n *=* *6; *Il17^−/−^*/CD, *n *=* *6; *Il17^+/+^*/MCD, *n *=* *6; *Il17^−/−^*/MCD, *n *=* *6). (A) Representative H&E-stained (upper panel) and Oil Red O-stained (lower panel) liver sections from different groups. Scale bar, 50 μm. Histological scores of liver tissue from the four groups described above. (B and C) Body weights and liver/weight ratios of the mice. (D) TAG levels in the liver. (E) Serum levels of ALT and AST. Significance versus *Il17*^+/+^ mice fed an MCD diet: NS, not significant; *, *P* < 0.05; **, *P* < 0.01; ***, *P* < 0.001.

Next, we observed the influence of IL-17 deletion on NAFLD/NASH progression. When the *Il17^+/+^* or *Il17^−/−^* mice were fed a CD, no obvious lipid accumulation or infiltration of immune cells in the liver was found (Fig. 2A). Histological examination showed that *Il17^−/−^* mice exhibited more lipid accumulation and inflammatory foci in the liver (Fig. 2A). Notably, *Il17^+/+^* and *Il17^−/−^* mice fed an MCD diet rapidly lost weight, but no significant differences were detected (Fig. 2B). However, *Il17^−/−^* mice had a lower liver-to-body weight ratio than *Il17^+/+^* mice, suggesting that IL-17 deletion is a risk factor for NAFLD/NASH development (Fig. 2C). Consistent with these histological findings, the liver levels of triglycerides (TAGs) were significantly increased in *Il17^−/−^* mice compared with those in *Il17^+/+^* mice (Fig. 2D). Furthermore, hepatocyte damage, as well as serum alanine aminotransferase (ALT) and aspartate aminotransferase (AST) levels, was more prominent in *Il17^−/−^* mice than in *Il17^+/+^* mice (Fig. 2E). Altogether, these data suggest that deletion of IL-17 promotes the progression of NAFLD/NASH.

### Influence of IL-17 on gut homeostasis.

**FIG 3:**
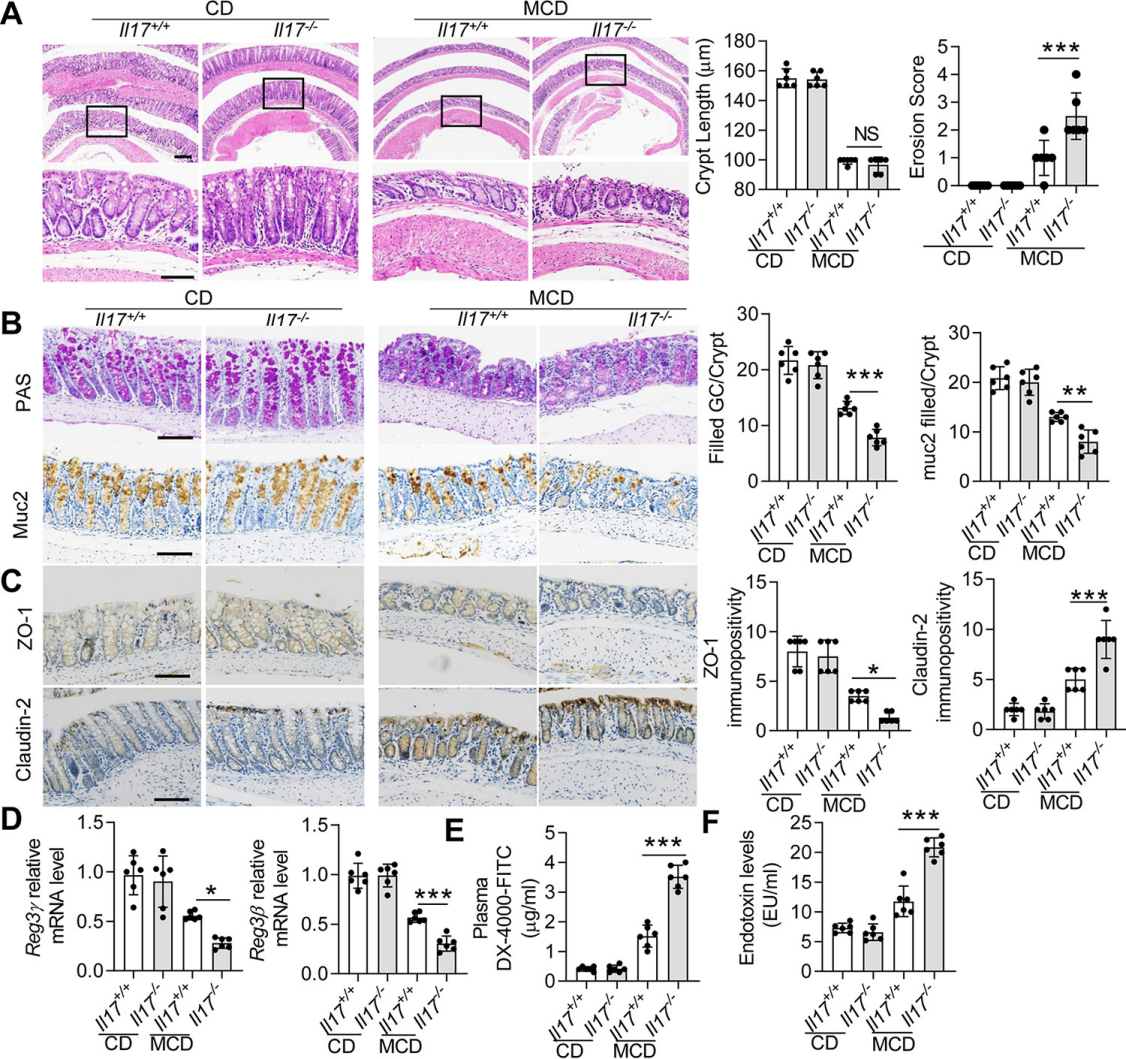
*Il17a* deletion increases intestinal permeability and endotoxin levels. The *Il17^+/+^* and *Il17^−/−^* mice were fed a CD or an MCD diet for 3 weeks. (A) Representative H&E-stained colon sections. The straight line indicates the extent of submucosal edema. Upper scale bar, 200 μm; lower scale bar, 50 μm. Quantification of the colon crypt length (left panel) and erosion score (right panel). (B) PAS staining (upper panel) and immunohistochemical staining for Muc2 (lower panel). Scale bar, 50 μm. (C) Immunohistochemical staining for ZO-1 (upper panel) and Claudin-2 (lower panel) in mice. Scale bar, 50 μm. (D) The mRNA levels of *Reg3*γ and *Reg3*β in the colon were determined by qPCR. The relative mRNA levels were normalized to those of *Gapdh*. (E) Intestinal permeability was assessed in *Il17^+/+^* and *Il17^−/−^* mice fed a CD or an MCD diet by measuring FITC-dextran levels in the serum 4 h after oral FITC-dextran administration. (F) Plasma LPS concentrations (EU/mL) were determined by enzyme-linked immunosorbent assay (ELISA). The data are expressed as means ± SD. Significance versus *Il17*^+/+^ mice fed an MCD diet: NS, not significant; *, *P* < 0.05; **, *P* < 0.01; ***, *P* < 0.001.

Endotoxemia usually occurs in NAFLD by increasing intestinal permeability (12), and intestinal IL-17 mediates the function of the intestinal barrier (21). Therefore, we next investigated whether IL-17 influences intestinal permeability during the consumption of an MCD diet. Hematoxylin and eosin (H&E) staining revealed that the intestinal (colon and SI) crypts were shorter in the MCD diet-fed mice, but no significant difference was observed between *Il17^+/+^* and *Il17^−/−^* mice (Fig. 3A; see Fig. S5A in the supplemental material). Furthermore, erosive epithelia were observed in the colons of MCD diet-fed mice, and the erosion score was significantly higher in the *Il17^−/−^* group (Fig. 3A); however, erosive epithelia were not evident in the SI (Fig. S5A). Histological examination of the colon and SI showed no obvious inflammatory infiltrates in CD- or MCD diet-fed mice; however, MCD diet-fed mice showed signs of early inflammation submucosal edema in the colon (Fig. 3A; Fig. S5A). The *Il17^−/−^* group demonstrated similar signs of inflammation, although to a higher degree (Fig. 3A). Furthermore, periodic acid-Schiff (PAS) and mucin-2 (Muc2) staining revealed fewer cells in the colonic mucosa of MCD diet-fed *Il17^−/−^* mice than that of MCD diet-fed *Il17^+/+^* mice (Fig. 3B). However, we did not observe a decline in goblet cells in the SI (Fig. S5B). These results indicated that IL-17 deletion might influence the function of the colon barrier.

10.1128/mbio.03688-21.6 FIG S5 *Il17a* deletion does not significantly alter the SI mucosa. (A) Representative H&E-stained SI sections. Scale bar, 100 μm. (B) PAS staining and immunohistochemical staining for Muc2. Scale bar, 50 μm. NS, not significant versus *Il17^+/+^* mice fed an MCD diet. Download FIG S5, TIF file, 2.7 MB. Copyright © 2022 He et al. 2022 He et al. https://creativecommons.org/licenses/by/4.0/ This content is distributed under the terms of the Creative Commons Attribution 4.0 International license.

ZO-1 is a key marker in maintaining intestinal integrity; however, Claudin-2, which forms cation-selective pores and makes the epithelial barrier more permissive (22), is highly expressed in the colonic enterocytes of mice fed an HFD (23). ZO-1 levels were reduced in the colonic enterocytes of *Il17^+/+^* mice fed an MCD diet compared with those of *Il17^+/+^* mice fed a CD diet (Fig. 3C). Claudin-2 was highly expressed in *Il17^+/+^* mice fed an MCD diet (Fig. 3C). However, compared with the *Il17^+/+^* mice fed an MCD diet, *Il17^−/−^* mice fed an MCD diet exhibited a greater reduction in ZO-1 and a greater increase in Claudin-2 in the colon (Fig. 3C). IL-17 is required to express C-type lectin regenerating protein 3γ (REG3γ) and REG3β in the colon (4, 24). Our data suggested that *Reg3*γ and *Reg3*β mRNA levels were decreased in *Il17^−/−^* mice compared with those in *Il17^+/+^* mice after MCD diet feeding (Fig. 3D). We also assessed the plasma levels of DX-4000–fluorescein isothiocyanate (FITC) in *Il17^+/+^* and *Il17^−/−^* mice. The plasma DX-4000–FITC concentration was obviously increased in *Il17^+/+^* mice fed an MCD diet compared with that in *Il17^+/+^* mice fed a CD diet and was significantly increased in *Il17^−/−^* mice fed an MCD diet compared with that in *Il17^+/+^* mice fed an MCD diet (Fig. 3E). In accordance with the plasma DX-4000–FITC levels, the endotoxin levels were obviously increased in *Il17^+/+^* mice fed an MCD diet compared with those in *Il17^+/+^* mice fed a CD and were significantly increased in MCD diet-fed *Il17^−/−^* mice compared with those in MCD diet-fed *Il17^+/+^* mice (Fig. 3F). These results further confirm that IL-17 is necessary for intestinal barrier integrity.

### IL-17 deficiency induces changes in the gut microbiota of MCD diet-fed mice.

**FIG 4:**
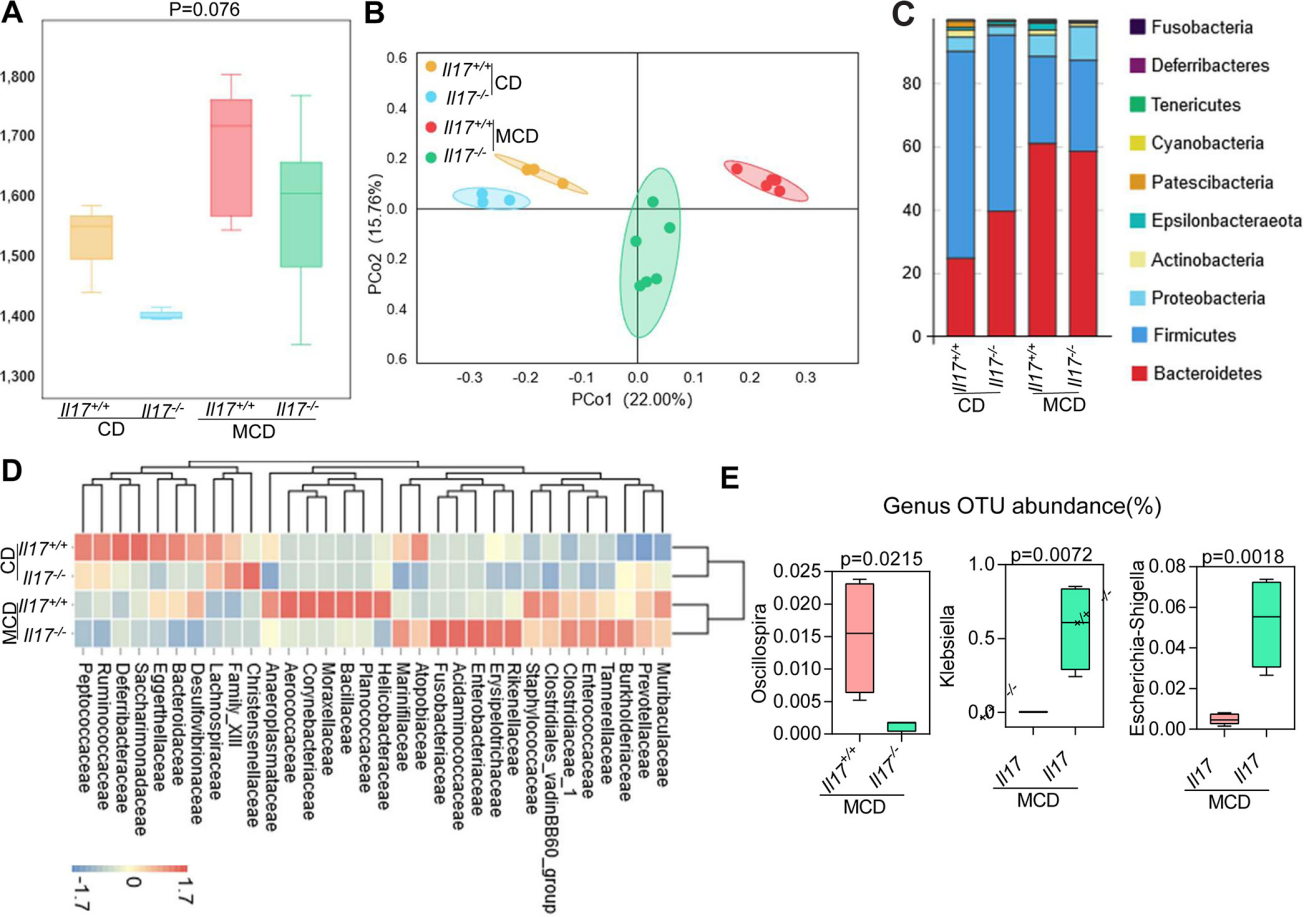
*Il17* modulates the intestinal microbiota. *Il17^+/+^* and *Il17^−/−^* mice were fed a CD or an MCD diet for 3 weeks. (A) Microbial richness in *Il17^+/+^* and *Il17^−/−^* mice fed a CD or an MCD diet based on the Chao1 index. (B) PCoA of *Il17^+/+^* and *Il17^−/−^* mice fed a CD or an MCD diet. (C) Relative abundance of operational taxonomic units (OTUs) at the phylum level in the gut microbiota of all groups. (D) Heat map analysis of effect size was performed to identify the differential abundances of bacteria between the different groups. (E) Relative abundance of OTUs of the genera *Oscillospira*, Klebsiella and Escherichia*-Shigella*.

An MCD diet modulates the gut microbiota (25). To evaluate whether IL-17 modulates microbiota dysbiosis, we monitored changes in the gut microbiota by 16S rRNA gene sequencing. The alpha diversity of the total bacterial community was slightly decreased in *Il17^−/−^* mice compared with that in *Il17^+/+^* mice, regardless of whether the mice were fed the CD or MCD diet; however, no significant difference in evenness or richness was observed among the groups (Fig. 4A). As shown in the principal-coordinate analysis (PCoA) plots, although an evident distribution between *I17^−/−^* and *Il17^+/+^* mice was observed, this distribution was more distinct between *I17^−/−^* and *Il17^+/+^* mice after MCD diet feeding (Fig. 4B). We also evaluated differences in the relative abundance at the phylum level among the groups. According to previous reports (26), *Firmicutes* and *Bacteroidetes* are the most abundant phyla in healthy mice. Significant differences in gut microbiota were observed in mice fed an MCD diet compared with those fed a CD; the gut microbiota of the CD-fed mice was mainly dominated by *Firmicutes*, whereas the predominant phylum in the MCD diet-fed group was *Bacteroidetes*. Interestingly, the abundance of the phylum *Proteobacteria*, which is involved in the prevalence of pathogenic bacteria and LPS-producing bacteria, was significantly increased in the MCD diet-fed mice of both genotypes, but higher in the *I17^−/−^* mice than in the *Il17^+/+^* mice (Fig. 4C). At the family level, the relative abundances of *Enterococcaceae* and *Enterobacteriaceae* were higher in the *Il17^−/−^* mice fed an MCD diet than in MCD diet-fed *Il17^+/+^* mice, whereas the abundance of *Staphylococcaceae* was slightly lower (Fig. 4D). At the genus level, we analyzed differences in abundance among all mice fed an MCD diet. The abundance of a genus of beneficial microorganisms, *Oscillospira*, which is involved in short-chain fatty acid production, was decreased in *Il17^−/−^* mice, and the abundances of pathogenic bacteria of the genera Klebsiella and Escherichia*-Shigella*, were increased in *Il17^−/−^* mice compared with that in *Il17^+/+^* mice (Fig. 4E). Together, these results demonstrate that IL-17 can modulate specific components of the commensal microbiota in MCD diet-fed mice.

### IL-17 originating from T_H_17 cells is crucial for maintaining intestinal barrier integrity in an MCD diet-fed murine model.

**FIG 5:**
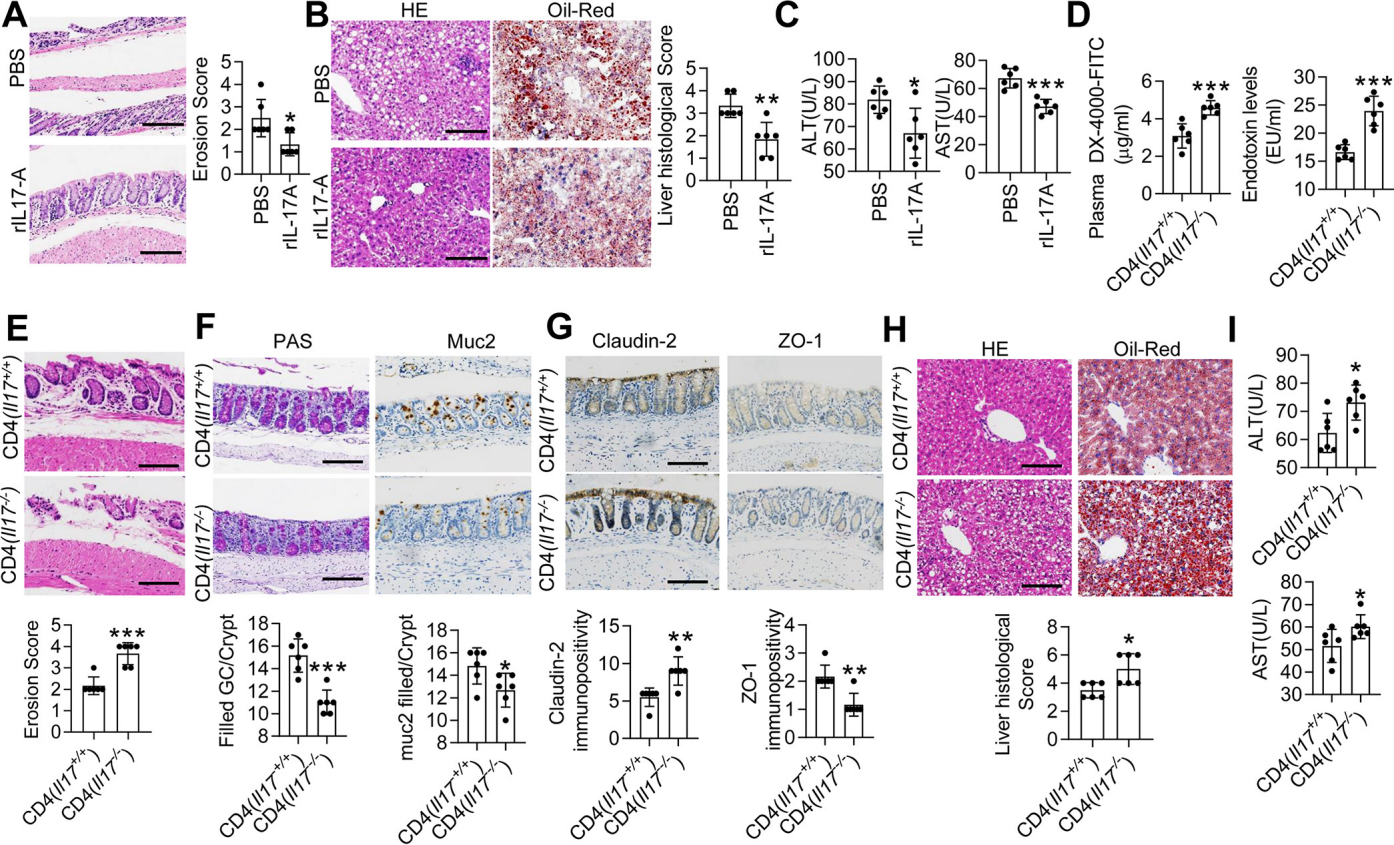
IL-17 in T cells rather than innate lymphoid cells maintains the intestinal barrier. *Il17^−/−^* mice were fed an MCD diet for 1 week and then were treated with PBS or rIL17A for the following 2 weeks. (A) Representative H&E-stained colon sections. Scale bar, 50 μm. Quantification of the erosion score. (B) Representative H&E-stained (left) and Oil Red O-stained (right) liver sections from PBS- and rIL17A- treated mice. Scale bar, 50 μm. Histological scores of liver tissue from the PBS and rIL17A groups. *Il17^−/−^* mice were fed an MCD diet for 1 week and then received CD4^+^ T cells originating from *Il17^+/+^* or *Il17^−/−^* mice. (C) Serum levels of ALT and AST. (D) The FITC-dextran levels in the mice that received CD4^+^ T cells from *Il17^+/+^* mice and those that received CD4^+^ T cells from *Il17^−/−^* mice were determined. Plasma LPS concentrations were determined by ELISA. (E) Representative H&E-stained colon sections. Scale bar, 50 μm. Quantification of the erosion scores. The straight line indicates the extent of submucosal edema. (F) PAS staining (left) and immunohistochemical staining for Muc2 (right) in the colon. Scale bar, 50 μm. (G) Immunohistochemical staining for Claudin-2 (left) and ZO-1 (right) in colon tissues. Scale bar, 50 μm. (H) Representative H&E-stained (left) and Oil Red O-stained (right) liver sections. Scale bar, 50 μm. Histological scores of liver tissue. (I) Serum levels of ALT and AST. The data are expressed as the means ± SD. Significance versus mice that received CD4^+^ T cells from *Il17*^+/+^ mice or PBS treatment: *, *P* < 0.05; **, *P* < 0.01; ***, *P* < 0.01.

To further confirm the relationship between IL-17 and intestinal permeability, *Il17^−/−^* mice were administered exogenous IL-17. The plasma DX-4000–FITC and serum LPS levels were decreased after recombinant IL-17A (rIL-17A) treatment (see Fig. S6A and B in the supplemental material). Histological examination of the colon revealed that rIL-17A treatment ameliorated the submucosal edema and intestinal erosion observed in the colons of MCD diet-fed *Il17^−/−^* mice (Fig. 5A). *Reg3*γ and *Reg3*β mRNA levels were also increased in the colon after rIL-17A treatment (Fig. S6C). The number of mucus-filled goblet cells and mucin-2 staining were also increased in the colon after rIL-17A treatment (Fig. S6D). Furthermore, analysis of Claudin-2 and ZO-1 expression revealed that rIL-17A treatment ameliorated the change in gut permeability by decreasing Claudin-2 expression and increasing ZO-1 expression (Fig. S6E). However, the histology, goblet cells, and Muc2 expression of SI were not significantly altered (see Fig. S7 in the supplemental material). Above all, hepatic steatosis, inflammatory cell infiltration, ALT, and AST were significantly attenuated after rIL-17A treatment (Fig. 5B and C).

10.1128/mbio.03688-21.7 FIG S6 rIL17A improves intestinal permeability and hepatic steatosis. *Il17^−/−^* mice were fed an MCD diet for 1 week and then were treated with PBS or rIL17A for the following 2 weeks. (A) Intestinal permeability assessed by serum FITC-dextran quantification at 4 h after oral administration in *Il17^−/−^* mice. (B) The plasma LPS concentrations were determined by ELISA. (C) The *Reg3*γ and *Reg3*β mRNA levels were determined by qPCR. The relative mRNA levels were normalized to that of *Gapdh*. (D) PAS staining (left) and immunohistochemical staining for Muc2 (right). Scale bar, 50 μm. (E) Immunohistochemical staining for Claudin-2 (left) and ZO-1 (right) in mice. Scale bar, 50 μm. The data are expressed as means ± SD. Significance versus the PBS group: *, *P* < 0.05; **, *P* < 0.01; ***, *P* < 0.01. Download FIG S6, TIF file, 2.0 MB. Copyright © 2022 He et al. 2022 He et al. https://creativecommons.org/licenses/by/4.0/ This content is distributed under the terms of the Creative Commons Attribution 4.0 International license.

10.1128/mbio.03688-21.8 FIG S7 Supplemental IL-17 does not significantly alter the SI mucosa. *Il17^−/−^* mice were fed an MCD diet for 1 week and then were treated with PBS or rIL17A for the following 2 weeks. (A) Representative H&E-stained SI sections. Scale bar, 100 μm. (B) PAS staining and immunohistochemical staining for Muc2. Scale bar, 50 μm. NS, not significant versus the PBS group. *Il17^−/−^* mice were fed an MCD diet for 1 week and then received CD4^+^ T cells originating from *Il17^+/+^* or *Il17^−/−^* mice. (C) Representative H&E-stained SI sections. Scale bar, 100 μm. (D) PAS staining and immunohistochemical staining for Muc2. Scale bar, 50 μm. NS, not significant versus the CD4^+^ (*Il17^+/+^*) group. Download FIG S7, TIF file, 2.7 MB. Copyright © 2022 He et al. 2022 He et al. https://creativecommons.org/licenses/by/4.0/ This content is distributed under the terms of the Creative Commons Attribution 4.0 International license.

IL-17 is secreted by many types of immune cells: among them, T_H_17 cells are regarded as the primary source of IL-17 (27). To further confirm that T_H_17 cells are related to the maintenance of intestinal barrier integrity and the development of NAFLD/NASH, CD4^+^ T cells isolated from *Il17^−/−^* mice and *Il17^+/+^* mice were transplanted into *Rag1^−/−^* mice. Both plasma DX-4000–FITC and serum LPS concentrations were increased in mice that received *Il17^−/−^* CD4^+^ T cells compared with those that received *Il17^+/+^* CD4^+^ T cells (Fig. 5D). Additionally, the recipient mice that received *Il17^−/−^* CD4^+^ T cells exhibited more severe intestinal barrier disturbance (Fig. 5E to G). Furthermore, both hepatic steatosis and the infiltration of inflammatory cells were attenuated in mice that received *Il17^+/+^* CD4^+^ T cells, as determined by histological analysis (Fig. 5H), and the levels of ALT and AST also decreased (Fig. 5I). These results demonstrate that IL-17 derived from T_H_17 cells is crucial to improve intestinal barrier function in an MCD diet-fed murine model.

### FMT improves gut permeability in MCD diet-fed *Il17^−/−^* mice.

**FIG 6:**
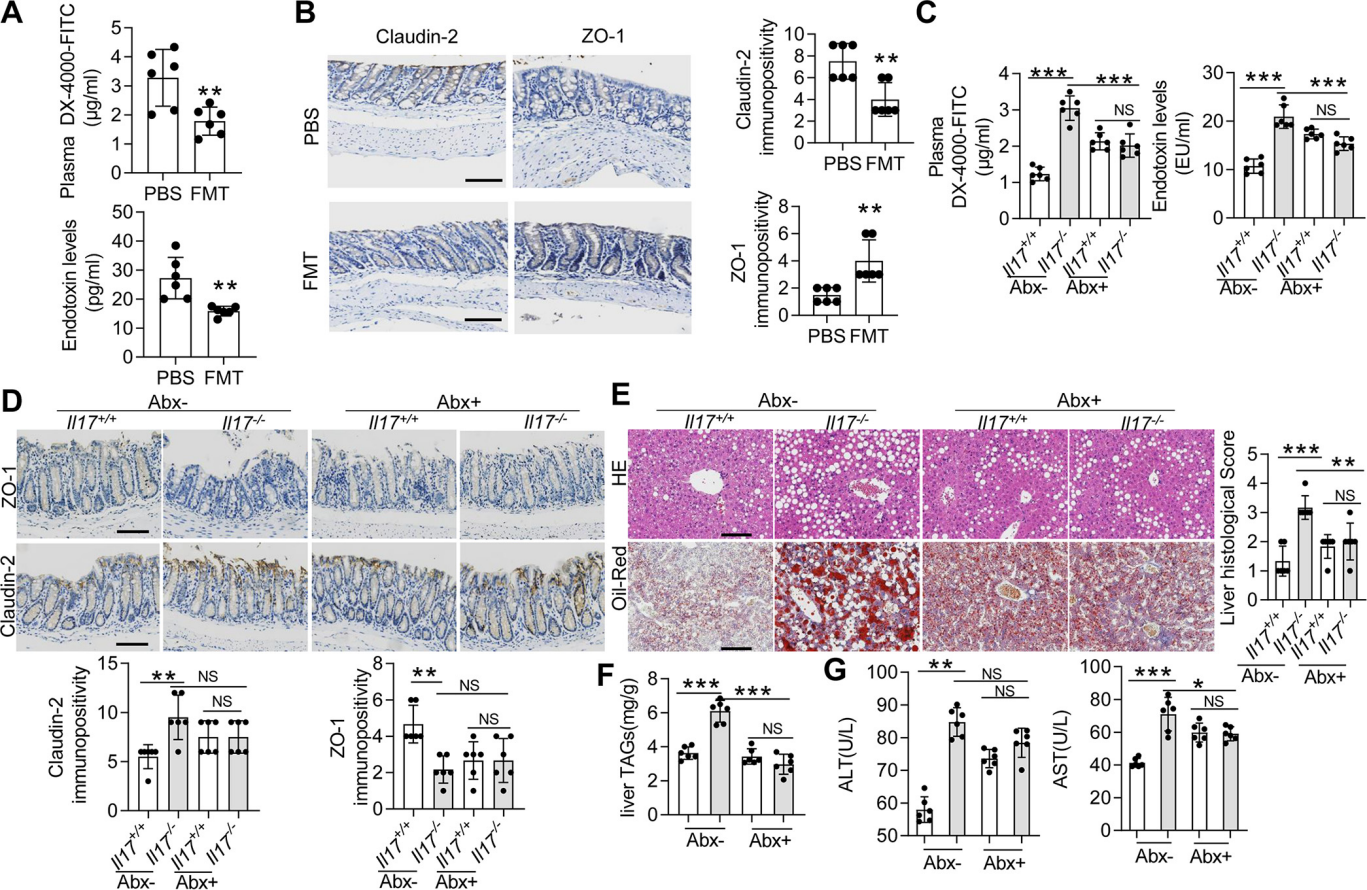
The gut microbiota promotes high intestinal permeability and hepatic steatosis in *Il17^−/−^* mice. After a week of adaptation to an MCD diet, *Il17^−/−^* mice were subjected to FMT or were inoculated with PBS by enema. (A) The FITC-dextran levels (upper panel) and plasma LPS concentrations (lower panel) were determined. (B) Immunohistochemical staining for Claudin-2 (left) and ZO-1 (right) in colon sections. Scale bar, 50 μm. The data are expressed as means ± SD. Significance versus the PBS-treated group: *, *P* < 0.05; **, *P* < 0.01; ***, *P* < 0.001. *Il17^−/−^* and *Il17^+/+^* mice were treated with or without Abx for 4 weeks and then were fed an MCD diet for 3 weeks (*n *=* *5 in each group). (C) FITC-dextran levels (upper panel) and plasma LPS concentrations (lower panel) were determined. (D) Immunohistochemical staining for ZO-1 (upper panel) and Claudin-2 (lower panel). Scale bar, 50 μm. (E) Representative H&E-stained (upper panel) and Oil Red O-stained (lower panel) liver sections. Scale bar, 50 μm. (F) TAG levels in the liver. (G) Serum levels of ALT and AST. The data are expressed as means ± SD. NS, not significant; *, *P* < 0.05; **, *P* < 0.01; ***, *P* < 0.001.

To determine whether IL-17 maintains intestinal barrier integrity by modulating the gut microbiota, *Il17^−/−^* mice were fed an MCD diet and then subjected to FMT, in which the donor was wild-type mice. The results of beta diversity analysis showed that the beta diversity of the *Il17^−/−^* mice treated with FMT differed from that of the *Il17^−/−^* mice treated with phosphate-buffered saline (PBS) (Fig. S8A). FMT treatment increased the abundance of *Bifidobacteriaceae* and decreased the abundances of *Enterococcaceae*, *Desulfovibrionaceae*, *Helicobacteraceae*, and *Enterobacteriaceae* (Fig. S8B). The LPS level and plasma concentration of DX-4000–FITC were decreased in the FMT group compared with those in the PBS group (Fig. 6A). H&E staining showed that the histological appearance of the colon was improved after FMT treatment (Fig. S8C), and PAS and Muc2 staining further confirmed that FMT improved barrier function (Fig. S8D). Similar results were obtained for the expression of Claudin-2 and ZO-1 (Fig. 6B). We then assessed liver pathology after FMT treatment. The liver-to-body weight ratio of the FMT group was increased compared with that of the PBS group (Fig. S8E). Histological examination showed that FMT treatment attenuated lipid accumulation and inflammatory foci in the liver (Fig. S8F). Additionally, the liver levels of TAGs and serum levels of ALT and AST also decreased after FMT treatment (Fig. S8G and H). Therefore, donors from wild-type mice could strengthen the colonic barrier integrity and weaken the NAFLD/NASH process in *Il17^−/−^* mice.

10.1128/mbio.03688-21.9 FIG S8 *Il17^−/−^* mice treated with FMT exhibit an improved intestinal erosive epithelium and hepatic steatosis. After a week of adaptation to an MCD diet, *Il17^−/−^* mice were subjected to FMT or treated with PBS by enema. (A) PCoA of *Il17^−/−^* mice subjected to FMT or treated with PBS. (B) Heat map analysis of the effect size to identify the differential abundances of bacteria. (C and D) Representative H&E- and PAS-stained colon sections and immunohistochemical staining for Muc2. Scale bar, 50 μm. (E) Liver/weight ratios. (F) Representative H&E-stained (left) and Oil Red O-stained (right) liver sections. Scale bar, 50 μm. (G) TAG levels in the liver. (H) Serum levels of ALT and AST. The data are expressed as means ± SD. Significance versus the PBS group: **, *P* < 0.01; ***, *P* < 0.001. Download FIG S8, TIF file, 0.3 MB. Copyright © 2022 He et al. 2022 He et al. https://creativecommons.org/licenses/by/4.0/ This content is distributed under the terms of the Creative Commons Attribution 4.0 International license.

### Abx improves gut permeability in MCD diet-fed *Il17^−/−^* mice.

To more directly investigate whether the observed phenotypic aggravation was due to the microbial communities in the gut, we pretreated *Il17^−/−^* mice and *Il17^+/+^* mice with antibiotics (Abx). Validation of the successful depletion of the microbiota was performed by quantitative PCR (qPCR). Because of epithelial cell shedding, we chose host DNA (mouse pIgR genomic region) as an internal control (28). All the mice subjected to Abx treatment exhibited a significantly reduced abundance of 16S rRNA of *Firmicutes*, *Bacteroidetes*, and *Epsilonproteobacteria* (see Fig. S9A and B in the supplemental material). However, the abundance of *Betaproteobacteria* was significantly increased, and those of Deltaproteobacteria and *Gammaproteobacteria* did not change (Fig. S9B). Next, we assessed whether microbiota depletion influences the process of NAFLD/NASH and found that the plasma concentration of DX-4000–FITC and serum LPS levels were increased in *Il17^+/+^* mice treated with Abx compared with those in untreated *Il17^+/+^* mice (Fig. 6C); however, the DX-4000–FITC and LPS levels were significantly decreased in *Il17^−/−^* mice treated with Abx (Fig. 6C). Accordingly, differences in Claudin-2 and ZO-1 expression were not observed between Abx-treated *Il17^−/−^* mice and Abx-treated *Il17^+/+^* mice (Fig. 6D). No significant differences in liver histology, liver levels of TAGs, or serum levels of ALT and AST were observed between the Abx-treated *Il17^−/−^* mice and Abx-treated *Il17^+/+^* mice (Fig. 6E to G). Altogether, our results confirmed increased intestinal permeability in *Il17^−/−^* mice fed an MCD diet because of changes in the gut microbiota.

10.1128/mbio.03688-21.10 FIG S9 Abx intervention significantly decreases the abundance of bacteria. *Il17^−/−^* and *Il17^+/+^* mice were treated with or without Abx for 4 weeks and then were fed an MCD diet for 3 weeks. (A) Microbiota genetic DNA was extracted from feces, and qPCR was used to analyze the level of 16S rRNA genes (V2 and V6 regions), relative to the level of the mouse pIgR genomic region. (B) *Firmicutes*, *Proteobacteria*, *Bacteroidetes*, *Betaproteobacteria*, *Epsilonproteobacteria*, Deltaproteobacteria, and *Gammaproteobacteria* were assessed based on 16S rRNA by qPCR. The data are expressed as means ± SD. *, *P* < 0.05; **, *P* < 0.01; ***, *P* < 0.001; NS, not significant. Download FIG S9, TIF file, 0.6 MB. Copyright © 2022 He et al. 2022 He et al. https://creativecommons.org/licenses/by/4.0/ This content is distributed under the terms of the Creative Commons Attribution 4.0 International license.

### Fecal transplantation from *Il17^−/−^* mice increased intestinal permeability in recipient mice.

**FIG 7:**
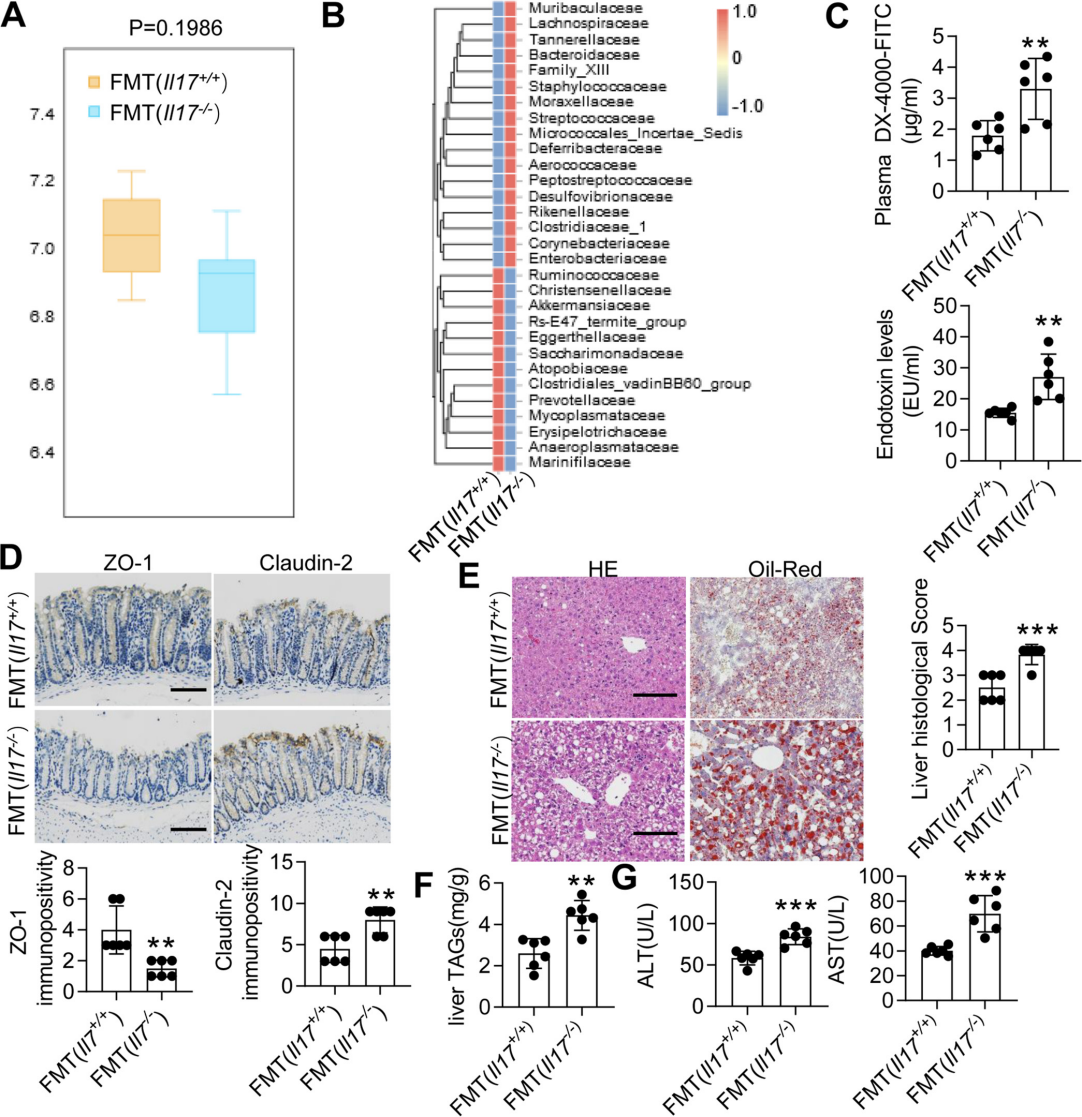
The gut microbiota that originated from *Il17^−/−^* mice increases intestinal permeability. After 6-week-old WT mice were treated with Abx for 4 weeks, these microbiota-depleted mice underwent FMT of feces originating from *Il17^−/−^* or *Il17^+/+^* mice. (A) PCoA of *Il17^−/−^* mice subjected to FMT from *Il17^−/−^* mice or FMT from *Il17^+/+^* mice. (B) Heat map analysis of the effect size was performed to identify the differential abundances of bacteria. (C) The FITC-dextran levels (left) and plasma LPS concentrations (right) were determined. (D) Immunohistochemical staining for ZO-1 (left) and Claudin-2 (right). Scale bar, 50 μm. (E) Representative H&E-stained (left) and Oil Red O-stained (right) liver sections. Scale bar, 50 μm. Each histological score is expressed as means ± SD. **, *P* < 0.01 versus the FMT (*Il17^+/+^*) group. (F) TAG levels in the liver. (G) Serum levels of ALT and AST. The data are expressed as means ± SD. Significance versus the FMT (*Il17*^+/+^) group: *, *P* < 0.05; **, *P* < 0.01, ***, *P* < 0.001.

To confirm whether the change in the gut microbiota in the *Il17^−/−^* mice directly contributed to increased gut permeability, we transplanted the microbiota of *Il17^−/−^* mice and *Il17^+/+^* mice into microbiota-depleted mice. Here, 16S rRNA gene sequencing analysis revealed no significant difference in the evenness or richness among the groups transplanted with the microbiota of *Il17^−/−^* and *Il17^+/+^* mice (Fig. 7A). However, at the family level, we observed increased abundances of *Staphylococcaceae*, *Enterobacteriaceae*, and *Streptococcaceae* in the *Il17^−/−^* FMT group (Fig. 7B). Additionally, one beneficial family of microorganisms, *Akkermansiaceae*, was decreased in the *Il17^−/−^* FMT group (Fig. 7B). Next, we examined gut permeability. Unlike transplantation of the microbiota of the *Il17^+/+^* mice, transplantation of the microbiota of the *Il17^−/−^* mice significantly increased the plasma concentration of DX-4000–FITC and serum LPS levels (Fig. 7C). Similarly, the decrease in ZO-1 expression was modestly improved, and the increased expression of Claudin-2 was reduced in mice that received the microbiota of *Il17^−/−^* mice (Fig. 7D). Histological analysis of the liver showed that the *Il17^−/−^* mouse microbiota aggravated lipid accumulation and the infiltration of immune cells (Fig. 7E). The liver levels of TAGs and serum levels of ALT and AST in mice that received the microbiota of the *Il17^−/−^* mice were also increased compared with those in mice that received the microbiota of *Il17^+/+^* mice (Fig. 7F and G). Altogether, our results showed that IL-17 modulated the gut microbiota and protected the MCD diet-fed model by improving intestinal barrier function.

## DISCUSSION

SFB are sufficient to induce T_H_17 cells and influence IL-17 and IL-22 levels (19), and IL-17 contributes to intestinal barrier function by influencing the expression of antimicrobial peptides (29). Additionally, IL-17 influences obesity and related metabolic disorders by shaping gut microbes, as reported in an HFD mouse model (7). Our data suggest that IL-17 originates from T_H_17 cells but not other cell types and plays a protective role in maintaining the balance between the intestinal barrier and gut microbiota, which is closely related to the development of NAFLD/NASH. Furthermore, we confirmed that IL-17 deficiency could disturb the gut microbiota composition in MCD diet-fed mice, as indicated by the increased abundance of pathogenic bacteria and decreased probiotics, exacerbating hepatic steatosis accumulation and promoting NAFLD/NASH progression.

NAFLD is usually found in obesity but also occurs in nonobese individuals. Host genetics, dietary composition, and other environmental factors can be associated with nonobese individuals (30). The gut microbiota is a nonnegligible factor in elucidating the pathogenesis of NAFLD, and some studies have shown that notable microbiome changes (including *Veillonellaceae* and *Ruminococcaceae*) are related to liver fibrosis found in nonobese subjects, but not in obese subjects (7). Herein, the changes in intestinal microbiota may further uncover the difference between nonobese and obese NAFLD. Diet-induced models, such as the MCD and HFD models, have long been used and have different advantages in eliciting an NAFLD phenotype; the HFD model typically resembles human NAFLD (31, 32). However, HFD models exhibit less severe NAFLD (31). The MCD diet model has the advantage of eliciting an unequivocal and severe NASH phenotype in a limited time frame. However, these models fail to induce metabolic comorbidities (31). IL-17 shapes the gut microbiota and is related to NAFLD in an HFD mouse model (7), but how IL-17 affects HFD-induced NAFLD via gut microbiota remains elusive. In the current study, an MCD diet murine model was used to reveal the function of host IL-17 in gut dysbiosis involved in the evolution of NAFLD to NASH. First, we analyzed the CD4^+^ T_H_ cell proportion in the liver and gut in the presence of MCD. Despite the increase in T_H_1 cells in the gut, the MCD diet did not alter liver T_H_1 cells, which are considered pathogenic. In contrast, Garidou et al. found no differences in the number of T_H_1 cells in the SI of mice after HFD feeding, but the change in T_H_1 cells in the liver and gut is a dynamic process (4). The composition and duration of the diets and animal housing conditions may have induced these discrepancies in T_H_ cell subsets (6).

In our study, the IL-17 levels were also decreased in the SI but not in the colon. These results further confirm that the proportion of T_H_17 cells in the SI in MCD diet-fed mice was decreased, indicating that SI T_H_17 cells respond similarly to the MCD diet and HFD to induce NAFLD, even at different stages. Despite a reduction in T_H_17 cells in the SI LP, the MCD diet did not alter the total number of T_H_17 cells in the colon LP, which was slightly decreased in HFD models (4). Although the causes of the discrepancies in T_H_17 cell alterations are unclear, several factors, including the housing conditions, composition of the diet, and methods of leukocyte isolation, may contribute to those alterations. However, whether IL-17 has a protective effect on the intestinal barrier after MCD diet feeding has not been evaluated. Therefore, we primarily assessed the intestinal barrier and plasma LPS levels by comparing MCD diet-fed *Il17^−/−^* mice with MCD diet-fed *Il17^+/+^* mice. Intestinal barrier injury was aggravated, and the severity of NAFLD/NASH, intestinal permeability, and endotoxemia were exacerbated in IL-17-deficient mice. We next confirmed that IL-17 derived from T_H_17 cells is crucial to regulate the gut microbiota balance and intestinal barrier integrity. These data indicate that the protective role of IL-17 in maintaining the intestinal barrier perhaps outweighs its tissue-damaging potential in NAFLD/NASH, similar to the conditions in IBD (27). Nevertheless, an important insight is that loss of intestinal barrier integrity can allow viable enteric commensal bacteria to translocate and colonize in extraintestinal organs, including liver, spleen, and mesenteric veins (33, 34). In addition, recent work found that Enterococcus gallinarum translocated to the mesenteric lymph nodes (MLNs), spleen, and liver, which promoted Th17 responses in autoimmunity (34–36). Future work is needed to identify whether live bacteria translocate to extraintestinal tissues in Il17^−/−^ mice, especially in liver.

An infusion of LPS commonly occurs in diet-related NAFLD (37). Here, compared with *Il17^+/+^* mice, *Il17^−/−^* mice exhibited defective intestinal integrity, aberrant expression of tight junction proteins, and a decreased number of goblet cells. However, the administration of rIL-17 obviously reversed intestinal injury and cured NASH. Furthermore, exacerbation of endotoxemia by IL-17 deficiency was not observed in Abx-treated mice, because the LPS levels and intestinal barrier integrity in *Il17^−/−^* mice paralleled those in *Il17^+/+^* mice, indicating that the depletion of the microbiota by Abx might restore colonic mucosal integrity. Next, fecal transplantation experiments revealed that the gut microbiota of *Il17^−/−^* mice promoted intestinal barrier injury in microbiota-depleted mice. Therefore, we speculated that the inhibition of probiotic growth and promotion of the overgrowth of certain pathogenic bacteria may be responsible for the symptoms observed in *Il17^−/−^* mice.

We identified specific changes in the relative abundances of different taxa among the groups. The abundance of the phylum *Proteobacteria*, which includes various harmful Gram-negative bacteria, such as Escherichia spp. and *Desulfovibrio* spp., was increased in MCD diet-fed mice and further increased in *Il17^−/−^* mice. This phenomenon was consistent with the increased LPS levels. Additionally, we demonstrated that *Il17^−/−^* mice fed an MCD diet exhibited overgrowth of *Enterococcaceae* and *Enterobacteriaceae* and inhibited the growth of *Oscillospira*, which produces short-chain fatty acids (38). Klebsiella and Escherichia*-Shigella* induced the redistribution of tight junction proteins (38), and both of these groups were also increased in *Il17^−/−^* mice fed an MCD diet. In the FMT experiment, transplantation of the feces of *Il17^−/−^* mice to microbiota-depleted mice increased the abundances of *Staphylococcaceae*, *Enterobacteriaceae*, and *Streptococcaceae* and decreased the abundance of *Akkermansiaceae*, which is considered a protective family of bacteria in diabetes (39). Our results revealed that T_H_17 cells in the SI were decreased after mice were fed an MCD diet, and IL-17 ameliorated endotoxemia in MCD diet-fed mice by modulating the gut microbiota balance and intestinal barrier injury, restoring epithelial cell organization and barrier function. Further studies are required to determine the role of IL-17 in the gut microbiota and better understand the mechanism involved.

## MATERIALS AND METHODS

### Mice, mouse diets, and treatments.

Wild-type (WT) C57BL/6 mice (6 to 8 weeks old) were purchased from Guangdong Medical Laboratory Animal Center (Guangzhou, China). Additionally, *Il17^−/−^* mice in a C57BL/6 background were provided by Chen Dong (Tsinghua University, China) (40), and *Rag1^−/−^* mice in a C57BL/6 background were purchased from the Model Animal Research Center of Nanjing University (Nanjing, China). All mice were maintained under specific-pathogen-free conditions at the Guangzhou Medical University Animal Experiment Center (Guangzhou, China), and the experimental procedures were approved by the Animal Ethics Committee of Guangzhou Medical University. All of the mice were fed either a methionine choline-sufficient diet (chow diet [CD]) (Research Diets, New Brunswick, NJ; catalogue no. A02082003BY) or an MCD diet (Research Diets; catalogue no. A02082003BR) for 3 weeks.

### Model 1.

To induce NAFLD, 4-week-old WT mice were acclimated for 2 weeks before being fed a CD or an MCD diet for 3 weeks and then were harvested at 9 weeks of age (*n *=* *6 per group). Liver and colon tissues were collected from the mice after 3 weeks of CD or MCD diet feeding. Serum was collected to determine the lipopolysaccharide (LPS) content.

### Model 2.

To induce an NAFLD model in mice in which *Il17* had been deleted, heterozygous mice (*Il17^+/−^*) were used for breeding. The pups were weaned such that *Il17^−/−^* littermate mice were cohoused with their WT littermates (*Il17^+/+^*). Mice were randomly separated from breeding cages at 6 weeks of age, allotted to different experimental groups (*Il17^+/+^*/CD, *n* = 6; *Il17^−/−^*/CD, *n* = 6; *Il17^+/+^*/MCD, *n* = 6; *Il17^−/−^*/MCD, *n* = 6) (41) and then fed a CD or an MCD diet for 3 weeks. The feces were collected for 16S rRNA gene sequencing.

### Model 3.

For recombinant IL-17A (rIL17A) intervention, 6-week-old *Il17^−/−^* mice were fed an MCD diet for 1 week. Next, the *Il17^−/−^* mice were divided into the phosphate-buffered saline (PBS) group and rIL17A group (where each mouse was intraperitoneally injected with 0.5 μg of mouse rIL17A [PeproTech, Rocky Hill, NJ] weekly in 0.1 mL of PBS for 2 weeks) at *n *=* *6 per group, and the mice were fed an MCD diet for an additional 2 weeks.

### Model 4.

Naïve CD4^+^ T cell purification and transfer were performed as previously described (42). For CD4^+^ T cell transfer studies, 6-week-old *Rag1^−/−^* mice were adapted to an MCD diet for 1 week, intraperitoneally injected with 5 × 10^6^ naive *Il17^−/−^* CD4^+^ T cells or naive *Il17^+/+^* CD4^+^ T cells, and then fed an MCD diet for another 2 weeks (*n *=* *6 per group).

### Model 5.

FMT was performed according to a previous study (43). After a week of adaptation to the MCD diet, 7-week-old recipient *Il17^−/−^* mice were inoculated with fresh transplant material or PBS (20 mg of feces/100 μL of PBS) by enema twice weekly for the following 2 weeks (*n *=* *6 per group). Enemas were performed as described in our previous study (44).

### Model 6.

For Abx treatment, a well-established cocktail of four broad-spectrum Abx (1 g/L of neomycin, 1 g/L of metronidazole, 1 g/L of ampicillin, and 0.5 g/L of vancomycin) (Sigma-Aldrich, St. Louis, MO, USA) was added to the drinking water of the mice. Six-week-old *Il17^−/−^* and *Il17^+/+^* mice were treated with or without Abx for 4 weeks and then fed an MCD diet for 3 weeks (*n *=* *6 per group). Abx was administered throughout the dietary feeding period.

### Model 7.

After the WT mice (6 weeks old) were treated with Abx for 4 weeks, the water containing Abx was replaced with regular water. The microbiota of the *Il17^−/−^* or *Il17^+/+^* mice was transplanted into microbiota-depleted mice (*n *=* *6 per group). Following transplantation, the mice were fed an MCD diet for another 2 weeks.

Additional methods can be found in Text S1 in the supplemental material.

10.1128/mbio.03688-21.1 TEXT S1 Supplemental materials and methods. Download Text S1, DOCX file, 0.03 MB. Copyright © 2022 He et al. 2022 He et al. https://creativecommons.org/licenses/by/4.0/ This content is distributed under the terms of the Creative Commons Attribution 4.0 International license.
