# Supplementary material for: Interleukin-17 Weakens the NAFLD/NASH Process by Facilitating Intestinal Barrier Restoration Depending on the Gut Microbiota
Source: mBio. 2022 Mar 10;13(2):e03688-21. doi: 10.1128/mbio.03688-21 (PMC9040850; doi:10.1128/mbio.03688-21)
Supplement: TEXT S1 [file mbio.03688-21-t0001.docx]

**Materials and methods**

**Intestinal permeability *in vivo***

*In vivo* intestinal permeability was measured as previously described [1]. Briefly, mice were fasted for 6 h and then administered 4-kDa fluorescent dextran-FITC (500 mg/kg, Sigma-Aldrich) by gavage. After 4 h, the mice were anaesthetized by sevoflurane, and the concentration of 4-kDa dextran-FITC in the serum was measured using a Varioskan Flash spectral scanning multimode reader (Thermo Fisher Scientific, Waltham, MA, USA) at an excitation wavelength of 485 nm and emission wavelength of 535 nm.

**Histological scoring**

The livers were excised, fixed in 4% paraformaldehyde, embedded in optimal cutting temperature (OCT) compound, cut into 7-μm slices and stained with Oil Red O (Sigma-Aldrich). The colon and liver tissue samples were fixed in 4% paraformaldehyde, embedded in paraffin, and cut into 4-μm sections for further analysis. The liver sections were stained with haematoxylin and eosin (H&E). The colon sections were stained with H&E and Periodic Acid Schiff (PAS) (Solarbio, Beijing, China). These procedures were performed according to the manufacturer’s protocols. The stained sections were imaged under a Leica DM6 microscope (Leica, Wetzlar, Germany).

The lesions in the liver sections were evaluated histologically according to the NASH CRN Scoring System as previously reported [2, 3]. The colon erosion scores were assigned as previously described as follows: 0, no erosion was detected; 1, 0-25% of the epithelium was eroded; 2, 25-50% of the epithelium was eroded; 3, 50-75% of the epithelium was eroded; 4, 75-100% of the epithelium was eroded; and 5, no epithelium was present [4]. The number of goblet cells/crypt was determined in 50 longitudinally sectioned crypt units. The crypt depth of the colon was measured by ImageJ software (National Institutes of Health, Bethesda, MD, USA). The investigator had special expertise in measuring lesions and was blinded to the treatment conditions of the mice.

**Mouse serum LPS and liver function analyses**

Commercial kits were used to measure alanine aminotransferase (ALT) and aspartate aminotransferase (AST) (Jiancheng Institute of Biotechnology, Nanjing, China) levels in the serum according to the manufacturer’s instructions. The LPS serum concentrations were determined using an ELISA kit (Cusabio Biotechnology Co., Ltd., Wuhan, China) according to the manufacturer’s instructions.

**Hepatic lipid analyses**

Commercial kits were used to measure triglycerides (TAG) (Jiancheng Institute of Biotechnology) levels in the liver according to the manufacturer’s instructions.

**Immunohistochemistry**

As previously described, different groups of mice were sacrificed, and the same colonic region in each mouse was fixed in 4% paraformaldehyde. Paraffin-embedded sections were deparaffinized and immunostained for Muc2 (Abcam, Cambridge, MA, USA), tight junction protein 1 (ZO-1) (Abcam) and Claudin-2 (Abcam). The stained sections were examined under a Leica DM6 microscope (Leica). Positive staining for Muc2, ZO-1 and Claudin-2 was semiquantified according to a previously described method [5]. The investigators examined the lesions in a blinded fashion.

**Quantitative real-time PCR**

Total RNA was isolated using TRIzol (Takara, Dalian, China) according to the manufacturer’s instructions. cDNA synthesis was performed using PrimeScript^TM^ RT Master Mix (Takara), and qPCR was performed using SYBR Green Master Mix (Takara). Real-time PCR was performed on a Bio-Rad CFX96 system (Bio-Rad Laboratories, CA, USA). The mRNA levels in the samples were normalized to the that of the housekeeping gene glyceraldehyde 3-phosphate dehydrogenase (*Gapdh*). mRNA analysis was performed using the comparative 2^-△△CT^ method.

Tissue from the distal SI was washed with ice-cold phosphate- buffered saline (PBS) and used for genomic DNA extraction. Tissue and faecal genomic DNA was extracted using the QiaAmp DNA Mini Kit (Qiagen, Valencia, CA, USA) according to the manufacturer’s instructions. To normalize the biopsy size, total bacteria (Eubacteria) were standardized with 18S from mice. The abundance of SFB was also measured by qPCR using group-specific 16S rDNA gene primers. The abundance of bacteria in the faeces was also measured by qPCR using host DNA (mouse pIgR genomic region), and other abundances were measured using bacterial 16S.

**16S rRNA gene analysis**

Mouse faecal bacterial DNA extraction, 16S rRNA gene PCR amplification and sequencing and 16S rRNA gene analysis were performed by Gene Denovo Biotechnology Company (Guangzhou, China). Faecal bacterial DNA was extracted from each sample, and the integrity and size of the DNA samples were determined by 1% agarose gel electrophoresis. The primers 341F (CCTACGGGNGGCWGCAG) and 806R (GGACTACHVGGGTATCTAAT) were used to amplify the V3–V4 region of the 16S rRNA gene. Sample preparation was performed as previously described [6]. The 16S rRNA gene sequences were analysed using operational taxonomic units (OTUs) selected with a threshold of 97% pairwise identity and classified taxonomically using the Ribosomal Database Project (RDP) classifier 2.0.1.

**Isolation of liver leukocytes and lymphocytes from the intestinal immune system**

Liver-infiltrating leukocytes and lymphocytes from the intestinal immune system were isolated as previously described [7, 8]. These lymphocytes were used for flow cytometry analysis.

**Cell staining and flow cytometry analysis**

Lymphocytes were isolated from the intestine and liver of mice. The cells were stained with fluorescence-conjugated antibodies from eBioscience (San Diego, CA, USA) (eFluor506-conjugated anti-mouse CD3, FITC-conjugated anti-mouse CD4, Percp-Cy5.5-conjugated anti-mouse interferon gamma (IFN-γ), APC-conjugated anti-mouse forkhead box protein 3 (FOXP3), PE-Cy7-conjugated anti-mouse T-bet, PE-conjugated anti-mouse RORγT, APC-conjugated anti-mouse γδ TCR and PE-Cy7-conjugated anti-mouse CD8a). Pacific blue-conjugated anti-mouse NK1.1 and BV421-conjugated anti-mouse IL17A were obtained from Biolegend (San Diego, CA, USA). For intracellular transcription factor staining, the cells were stimulated with Cell Stimulation Cocktail (plus protein transport inhibitors) (eBioscience) for 4 h and stained using a Cytofix/Cytoperm staining kit (BD Biosciences, San Diego, CA, USA). Flow cytometry was performed using a FACSVerse cytometer (BD Biosciences, San Jose, CA, USA), and data were subsequently analysed using FlowJo X (Tree Star, Ashland, OR, USA).

**Statistical analysis**

GraphPad Prism 8.0 software (GraphPad Software Inc., USA) was used for data analysis. Statistical analysis was performed using Student’s t test, and comparisons of more than two groups were performed by one-way ANOVA. P<0.05 was used to indicate statistical significance.

1. Cani PD, Possemiers S, Van de Wiele T, Guiot Y, Everard A, Rottier O, et al. Changes in gut microbiota control inflammation in obese mice through a mechanism involving GLP-2-driven improvement of gut permeability. Gut. 2009; 58: 1091-103.

2. Ye D, Yang K, Zang S, Lin Z, Chau HT, Wang Y, et al. Lipocalin-2 mediates non-alcoholic steatohepatitis by promoting neutrophil-macrophage crosstalk via the induction of CXCR2. Journal of hepatology. 2016; 65: 988-97.

3. Kleiner DE, Brunt EM, Van Natta M, Behling C, Contos MJ, Cummings OW, et al. Design and validation of a histological scoring system for nonalcoholic fatty liver disease. Hepatology. 2005; 41: 1313-21.

4. Lu P, Bar-Yoseph F, Levi L, Lifshitz Y, Witte-Bouma J, de Bruijn AC, et al. High beta-palmitate fat controls the intestinal inflammatory response and limits intestinal damage in mucin Muc2 deficient mice. PloS one. 2013; 8: e65878.

5. He S, Xue M, Liu C, Xie F, Bai L. Parathyroid Hormone-Like Hormone Induces Epithelial-to-Mesenchymal Transition of Intestinal Epithelial Cells by Activating the Runt-Related Transcription Factor 2. The American journal of pathology. 2018; 188: 1374-88.

6. Zhang J, Yang Y, Lei Z, Huang L, Yang F, Zhang N, et al. Antitumor Ability of Berberine Accompanied by Modulation of Gut Microbiome in Sarcoma-180 Tumor-bearing Mice. International Journal of Pharmacology. 2018; 14: 460-70.

7. Garidou L, Pomie C, Klopp P, Waget A, Charpentier J, Aloulou M, et al. The Gut Microbiota Regulates Intestinal CD4 T Cells Expressing RORgammat and Controls Metabolic Disease. Cell metabolism. 2015; 22: 100-12.

8. Qiu Z, Sheridan BS. Isolating Lymphocytes from the Mouse Small Intestinal Immune System. J Vis Exp. 2018.
